# Management of patients with biliary sphincter of Oddi disorder without sphincter of Oddi manometry

**DOI:** 10.1186/1471-230X-10-124

**Published:** 2010-10-22

**Authors:** Evangelos Kalaitzakis, Tim Ambrose, Jane Phillips-Hughes, Jane Collier, Roger W Chapman

**Affiliations:** 1 Department of Gastroenterology, John Radcliffe Hospital, OX3 9DU Oxford, UK; 2 Institute of Internal Medicine, Sahlgrenska Academy, University of Gothenburg, Magtam lab, Vita stråket 12, Sahlgrenska University Hospital, 413 45 Gothenburg, Sweden; 3 Department of Radiology, John Radcliffe Hospital, OX3 9DU Oxford, UK

## Abstract

**Background:**

The paucity of controlled data for the treatment of most biliary sphincter of Oddi disorder (SOD) types and the incomplete response to therapy seen in clinical practice and several trials has generated controversy as to the best course of management of these patients. In this observational study we aimed to assess the outcome of patients with biliary SOD managed without sphincter of Oddi manometry.

**Methods:**

Fifty-nine patients with biliary SOD (14% type I, 51% type II, 35% type III) were prospectively enrolled. All patients with a dilated common bile duct were offered endoscopic retrograde cholangiopancreatography (ERCP) and sphincterotomy whereas all others were offered medical treatment alone. Patients were followed up for a median of 15 months and were assessed clinically for response to treatment.

**Results:**

At follow-up 15.3% of patients reported complete symptom resolution, 59.3% improvement, 22% unchanged symptoms, and 3.4% deterioration. Fifty-one percent experienced symptom resolution/improvement on medical treatment only, 12% after sphincterotomy, and 10% after both medical treatment/sphincterotomy. Twenty percent experienced at least one recurrence of symptoms after initial response to medical and/or endoscopic treatment. Fifty ERCP procedures were performed in 24 patients with an 18% complication rate (16% post-ERCP pancreatitis). The majority of complications occurred in the first ERCP these patients had. Most complications were mild and treated conservatively. Age, gender, comorbidity, SOD type, dilated common bile duct, presence of intact gallbladder, or opiate use were not related to the effect of treatment at the end of follow-up (p > 0.05 for all).

**Conclusions:**

Patients with biliary SOD may be managed with a combination of endoscopic sphincterotomy (performed in those with dilated common bile duct) and medical therapy without manometry. The results of this approach with regards to symptomatic relief and ERCP complication rate are comparable to those previously published in the literature in cohorts of patients assessed by manometry.

## Background

Sphincter of Oddi disorder (SOD) is a syndrome involving recurrent abdominal pain, with or without abnormalities in liver or pancreatic chemistries or duct dilatation. The paucity of controlled data for the treatment of most biliary SOD types and the incomplete response to therapy seen in clinical practice as well as in several trials [1-3], has generated significant controversy as to what the best course of management of these patients is. Consensus opinion holds that patients with biliary SOD type I, presenting with abdominal pain, raised liver function tests, and dilated common bile duct (CBD), should be treated with papillary decompression by means of endoscopic or surgical sphincterotomy [4,5]. However, the appropriate management of patients with biliary SOD type II and III remains unresolved. Current recommendations, based on two randomized controlled trials, include the use of sphincter of Oddi manometry (SOM) in patients with SOD type II so that sphincterotomy may be performed only in those with elevated pressure [5]. The results of these trials, however, are not in agreement with those of several uncontrolled studies in which outcome was not found to be associated with SOM findings [6-8]. There is no firm evidence as regards to the patients with suspected biliary SOD type III who should be carefully evaluated and treated pharmacologically prior to considering SOM and sphincterotomy [5].

Suspected sphincter of Oddi dysfunction has been shown in several studies to be related to increased complication risk at endoscopic retrograde cholangiopancreatography (ERCP) and SOM/sphincterotomy [9-11]. Thus, the potential benefits should be weighed against this increased complication risk prior to any attempt to treat SOD with endoscopic sphincterotomy.

The aim of the current study was to evaluate the natural history of a series of patients with biliary SOD managed without SOM.

## Methods

### Patients

All consecutive patients diagnosed with biliary SOD who were reviewed in the outpatient clinic of the Department of Gastroenterology at the John Radcliffe Hospital in Oxford, UK during the period October 2003 - September 2008 were prospectively identified and enrolled in the study. The diagnosis of biliary SOD was established according to the Rome II [12] or, after 2006, the Rome III criteria [5]. Other causes of abdominal pain were excluded by means of endoscopic and imaging investigations as clinically indicated. All patients had had a trial of proton pump inhibitors for at least four weeks that had been ineffective. Transabdominal ultrasound and magnetic resonance cholangiopancreatography (MRCP) were performed on all patients. Patients were categorized, according to the modified Geenen-Hogan biliary classification, into type I (dilated CBD (≥6 mm on ultrasound or MRCP) and abnormal aspartate aminotransferase, alanine aminotransferase, bilirubin, or alkaline phosphatase > 2 times normal values on 2 or more occasions), II (dilated CBD or any of the previously mentioned laboratory abnormalities), or III (none of the previously mentioned laboratory or imaging criteria) [5]. Patients with pancreatic SOD alone, pancreas divisum, pancreaticobiliary malignancy, recurrent pancreatitis, or any other potential cause of abdominal pain identified during routine clinical investigations were excluded from the study. No patient had gallbladder or bile duct stones or sludge on imaging or ERCP. No patient had abnormal pancreas or pancreatic duct on imaging or raised pancreatic amylase.

### Management

**Figure 1:**
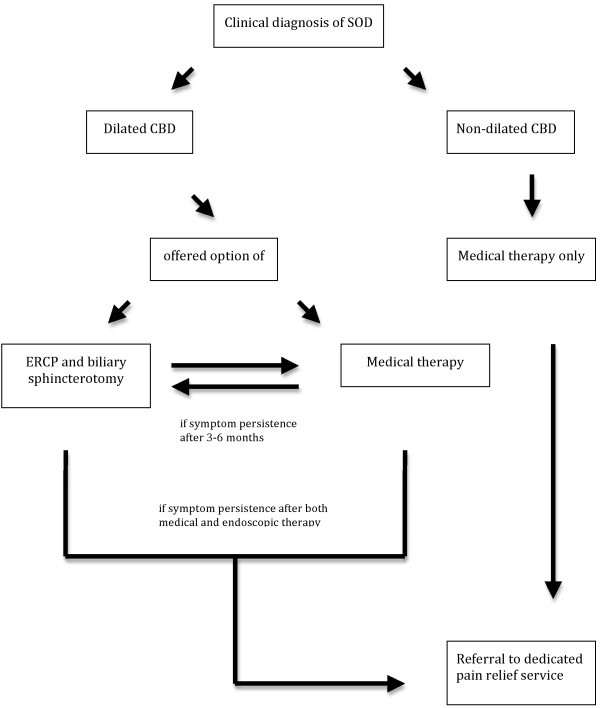
Flow chart of the management of the current cohort of patients with a clinical diagnosis of SOD (n = 59)

Following initial evaluation, patients with a clinical diagnosis of SOD type I and type II with dilated CBD were offered the option of biliary sphincterotomy or medical therapy. Medical treatment consisted of low dose amitryptiline (10-50 mg OD) followed by nifedipine (20 mg OD) [13] and glyceril trinitrate (GTN) spray. Regular pain relief medications such as paracetamol (500 mg 1-2 tablets QDS prn), tramadol (50-100 mg TDS prn), and codeine phosphate (30 mg 1-2 tablets QDS prn) were also used as needed alone or in combination with the previously mentioned medications. Patients with SOD type I and type II with a dilated CBD who initially opted for medical treatment but experienced symptom persistence after 3-6 months' follow-up were offered biliary sphincterotomy. Patients with SOD II with non-dilated CBD as well as patients with SOD III were offered medical treatment alone as described above. Patients with no satisfactory symptom relief after the above strategy were referred to a dedicated pain relief service. The management of patients with a clinical diagnosis of biliary SOD is summarized in Figure 1.

### Follow-up and data collection

Patient files were scrutinized and the following clinical data were extracted: age at diagnosis, gender, follow-up time (time from diagnosis until last outpatient clinic visit during the study period, until discharge or until last clinic visit before being lost to follow-up), imaging findings, treatment modalities used for SOD and their effect on symptoms as documented in the patient notes, comorbidities, and medications. In particular, the number of ERCPs performed in each patient case as well as the indication, any therapeutic procedures and their effect on patient symptoms, and any complications were registered. No prophylaxis by means of anti-proteasic drugs was given. Post-ERCP complications and their severity were defined as previously described [10]. The study protocol was approved by the Milton Keynes Research Ethics Committee and written informed consent was obtained by all patients.

### Statistics

Data are presented as median and interquartile range (IQR) or n and % as appropriate. The Mann-Whitney U test was performed for calculations of differences between groups. The Fisher's exact test was used for comparison between qualitative variables. All tests were two tailed and conducted at a 5% significance level.

## Results

Basic patient characteristics are shown in table 1. By the end of the study period, 14/59 patients (23%) were discharged from the outpatient clinic, 8/59 (14%) were lost to follow-up, and 37/59 (63%) were still under active follow-up. Overall, 33/59 (56%) of patients had an intact gallbladder.

**Table 1:** Basic characteristics of patients with biliary sphincter of Oddi disorder (n = 59)

Age at diagnosis (years)	46 (37-59)
Women/Men	53/6 (90%/10%)
Follow-up (months)	15 (6-35)
Previous cholecystectomy	26 (44%)
Biliary SOD type	
Type I	8 (14%)
Type II	30 (51%)
Dilated CBD	25 (42%)
Raised LFTs	5 (8%)
Type III	21 (35%)
Comorbidity^a^	39 (66%)
Other functional gastrointestinal disorder	16 (27%)
Psychiatric disease^b^	11 (19%)
Chronic liver disease^c^	10 (17%)
Analgesics upon initial assessment	23 (39%)
Opiates upon initial assessment^d^	21 (36%)

Data are presented as median and IQR or n and % as appropriate

^a^Patients who had other types of disease apart from the sphincter of Oddi disorder

^b^Eight patients out of 11 had depression and 3/11 anxiety

^c^Five patients out of 10 had non-alcoholic fatty liver disease, 2/10 primary biliary cirrhosis, 1/10 non-alcoholic steatohepatitis, 1/10 chronic hepatitis B, and 1/10 chronic hepatitis C

^d^Eleven out of 21 patients were receiving codeine, 5/21 tramadol, 3/21 morphine, 1/21 morphine and tramadol, and 1/21 codeine and tramadol

### Effect of medical and endoscopic therapy

The management and outcome of all patients with a clinical diagnosis of biliary SOD is shown in table 2. Overall, at last follow-up 9/59 patients (15.3%) reported complete symptom resolution, 35/59 (59.3%) improvement, 13/59 (22%) unchanged symptoms, and 2/59 (3.4%) symptom deterioration. Thirty out of 59 (51%) of patients experienced symptom resolution or improvement on medical treatment only, 7/59 (12%) after ERCP/sphincterotomy, and 6/59 (10%) after both medical treatment and ERCP/sphincterotomy. One patient experienced spontaneous symptom resolution with no particular treatment 6 months after presentation. Twelve patients out of 59 (20%) experienced at least one recurrence of symptoms (range 1-4) after initial response to medical and/or endoscopic treatment.

**Table 2:** Management and outcome of all patients with a clinical diagnosis of biliary SOD (n = 59)

	Follow-up (months)	Medical therapy	Endoscopic sphincterotomy	Symptom status at last follow-up				Recurrence
					
				Deterioration	Unchanged	Improvement	Resolution	
SOD I (n = 8)	12.5 (7-30)	7 (87.5%)	6 (75%)	1 (12.5%)	2 (25%)	3 (37.5%)	2 (25%)	2 (25%)
SOD II (n = 30)	24 (5-41)	28 (93%)	16 (53%)	0	7 (23%)	20 (67%)	3 (10%)	8 (27%)
dilated CBD (n = 25)	24 (13-40)	23 (92%)	16 (64%)	0	7 (28%)	15 (60%)	3 (12%)	7 (28%)
non-dilated CBD (n = 5)	28 (13-92)	5 (100%)	0	0	0	5 (100%)	0	1 (20%)
SOD III (n = 21)	12 (8-26)	21 (100%)	1 (5%)	1 (5%)	4 (19%)	12 (57%)	4 (19%)	2 (10%)
All patients (n = 59)	15 (6-35)	56 (95%)	23 (39%)	2 (3.4%)	13 (22%)	35 (59.3%)	9 (15.3%)	12 (20%)

### Medical interventions

In all, 21/59 (35.6%) patients experienced symptom resolution or improvement on low-dose tricyclic antidepressants (mainly amitryptiline, in two cases in combination with GTN spray and in 2 other cases in combination with tramadol), 3/59 (5%) on GTN spray, 2/59 on buscopan, 2/59 on morphine, and 1 each on nifedipine, low-dose citalopram, low-dose venlafaxine, diclofenac, paracetamol, tramadol, gabapentin (in combination with tramadol), and low-fat diet alone. Although 7/21 patients who were receiving opiates at baseline were weaned off these medications, another 6 patients were started on opiates (mainly tramadol) during the follow-up period. Thus, by the last clinic review 20/59 (34%) of patients were on opiates.

### ERCP and sphincterotomy

A total of 50 ERCP procedures were performed in 24/59 (41%) patients (range 1-7). Ten out of 24 patients (42%) had only one ERCP performed but the rest (14/24) had 2-7 procedures during the follow-up period. Indications for first ERCPs were intention to perform biliary sphincterotomy (CBD ≥ 6 mm on ultrasound or MRCP) in all patients apart from two who had an ERCP done as they could not undergo MRCP due to claustrophobia. They were both found to have dilated CBD and underwent sphincterotomy. A third patient who was classified as biliary SOD type III underwent ERCP and biliary sphincterotomy after careful discussion of the expected benefit and the risks of the procedure. No sphincterotomy or other intervention was performed in 1/24 of the patients undergoing ERCP on which CBD dilation previously seen on MRCP, was not confirmed. Thus, 23/24 patients underwent endoscopic sphincterotomy (table 3). In all, 14/23 patients (61%) undergoing ERCP/sphincterotomy reported initial symptom resolution/improvement as a result of the ERCP interventions. However, symptom recurrence/worsening after initial effect was reported by 9/14 (64%) during follow-up. Recurrence occurred after a mean of 13 months (median 18 months, range 1-24 months) following endoscopic sphincterotomy. Three out of 9 patients were treated with medications but further ERCP intervention was performed in 6/9 patients. Three out of 6 patients underwent pancreatic SOM and two were found to have raised pancreatic sphincter pressures and, thus, received pancreatic sphincterotomy. One of these recurred and was referred for transduodenal sphincteroplasty with good initial effect. Due to symptom recurrence during follow-up she required medical treatment leading to some pain relief (table 3, patient no 22). Another 3/6 patients underwent a trial of stenting. One was found to have re-stenosis (scarring at the biliary orifice not allowing the free passage of a small balloon (10-12 ml) and underwent further biliary sphincterotomy following a period of biliary stenting (table 3, patient no 19). The other two patients who underwent biliary stenting were found to have patent sphincterotomies (table 3, patients no 20 and 21). One responded and was thus referred for choledochojejunostomy with good initial effect. She recurred during further follow-up and required medical treatment leading to some pain relief (table 3, patient no 21). The other patient did not respond to biliary stenting and was referred to the pain relief team (table 3, patient no 20).

**Table 3:** Risk factors for post-ERCP pancreatitis and outcome of patients undergoing endoscopic sphincterotomy (n = 23).

Patient	Gender	Age at presentation	SOD type	Follow-up (months)	No of ERCPs	Pancreatic duct injection at 1st ERCP	Pre-cut sphincterotomy at 1st ERCP	Outcome of 1st ERCP	Recurrence after 1st ERCP	Complication
1	Female	49	I	50	4^a^	Yes	No	No effect		No
2	Female	66	II	89	1	Yes	Yes	No effect		pancreatitis
3	Male	34	III	0	1	Yes	No	No effect		pancreatitis
4	Female	29	II	37	2^b^	No	No	Improvement	Yes	No
5	Female	36	I	16	2	Yes	Yes	Resolution	No	pancreatitis (1st ERCP)
6	Female	27	I	10	1	No	No	Improvement	No	No
7	Female	44	II	27	1	Yes	Yes	No effect		retroperitoneal perforation
8	Female	47	II	39	2^c^	Yes	No	No effect		pancreatitis (2nd ERCP)
9	Female	49	II	24	1	No	Yes	Resolution	No	No
10	Female	44	II	22	1	No	No	Improvement	No	No
11	Female	46	II	56	2^d^	No	No	No effect		No
12	Female	49	II	43	2^e^	No	No	No effect		No
13	Female	47	II	60	4^f^	No	No	No effect		No
14	Female	26	I	13	1	No	No	Resolution	No	No
15	Female	46	II	15	1	No	No	No effect		No
16	Female	52	II	34	2^g^	Yes	No	Improvement	Yes	No
17	Female	57	II	28	1	No	No	Improvement	Yes	pancreatitis
18	Female	67	II	35	1	No	No	Resolution	Yes	No
19	Female	55	II	49	4^h^	Yes	No	Resolution	Yes	No
20	Female	46	II	92	3^i^	No	No	Resolution	Yes	pancreatitis (1st ERCP)
21	Female	27	II	110	7^j^	No	No	Improvement	Yes	No
22	Female	29	I	35	3^k^	Yes	No	Improvement	Yes	pancreatitis (3rd ERCP)
23	Female	43	I	2	2	No	Yes	Improvement	Yes	pancreatitis (1st ERCP)

Conventional over-the-wire biliary sphincterotomy was performed in all patients. In some patients, pre-cut sphincterotomy was performed to obtain access to the common bile duct prior to conventional sphincterotomy. The pre-cut and conventional sphincterotomy were performed during the same procedure apart from patient no 5 and 23 in whom they were performed a few weeks apart from each other as access to the common bile duct was achieved on a subsequent procedure a few weeks after pre-cut sphincterotomy.

^a^2nd ERCP, sphincterotomy extended; 3rd ERCP, trial of stent; 4th ERCP, stent removal as it was ineffective

^b^2nd ERCP for pancreatic sphincter of Oddi manometry showing raised pressure, pancreatic sphincterotomy performed leading to symptom improvement

^c^2nd ERCP, sphincterotomy assessed to be inadequate and was widened with no effect on symptoms

^d^2nd ERCP showed patent sphincterotomy, no endotherapy performed

^e^2nd ERCP for pancreatic SOM but pancreatic duct cannulation failed

^f^2nd ERCP done as pancreatic duct appeared dilated on follow-up MRCP. Pancreatic orifice appeared stenosed. No endotherapy performed as pancreatic stent could not be inserted despite deep guide wire pancreatic duct cannulation; 3rd ERCP for repeat attempt to perform pancreatic sphincterotomy, instrument failure during procedure; 4th ERCP pancreatic stenting and pancreatic sphincterotomy achieved leading to improvement in symptoms

^g^2n ERCP for pancreatic SOM normal pancreatic pressure thus no endotherapy performed

^h^2nd ERCP showed re-stenosed biliary sphincterotomy, biliary stenting performed; 3rd ERCP cholangitis due to stent obstruction, re-stenting; 4th ERCP extended sphincterotomy

^i^2nd ERCP showed patent sphincterotomy, biliary stenting performed; 3rd ERCP removal of stent as ineffective

^j^2nd ERCP, patent sphincterotomy, biliary stenting which was effective; 3rd-7th ERCP stent changes until surgery (choledochojejunostomy)

^k^2nd ERCP done for pancreatic sphincter of Oddi manometry, raised pancreatic pressure found and pancreatic sphincterotomy performed with improvement in symptoms; 3rd ERCP done due to symptom recurrence showed patent sphincterotomies, referred for surgery (open transduodenal sphincteroplasty)

### ERCP-related complications

All patients were routinely monitored as inpatients overnight post-ERCP. In all, a complication occurred in 9 out of 50 procedures (18%). There was no post-ERCP mortality. Eight patients had post-ERCP pancreatitis, 6/8 mild and 2/8 severe. Another patient had a retroperitoneal perforation with contrast being injected into the portal vein. She was treated conservatively as an inpatient for 6 days. A total of 7/9 (78%) complications occurred in the first ERCP each one of these patients had. One out of 9 complications occurred in a second and another in a third ERCP (pancreatitis in both cases). A total of 6 out of 23 (26%) patients who underwent endoscopic sphincterotomy experienced post-ERCP pancreatitis after their first ERCP.

### Factors related to symptom resolution/improvement during follow-up

Patients with symptom resolution/improvement had been followed-up for longer periods of time compared to patients with stable/deteriorated symptoms at the end of follow-up (23 months (11-39) vs. 3 months (0-12), p < 0.001). However, age at presentation, gender, comorbidity, other functional gastrointestinal disorder, psychiatric disease, liver disease, opiates upon initial assessment, or the presence of intact gallbladder were not found to be related to symptom resolution/improvement by the end of follow-up (data not shown). The presence of a dilated CBD was not more common among patients whose symptoms resolved or improved (52%) compared to those whose symptoms remained stable or deteriorated (67%) at the end of follow-up (p > 0.05). The proportions of patients reporting symptom resolution/improvement during follow-up did not differ significantly (p > 0.05) among patients with SOD type I (63%), type II (77%), or type III (76%). Among patients undergoing ERCP, the proportion of patients experiencing a post-ERCP complication did not differ significantly between patients reporting symptom resolution/improvement (35%) and those reporting stable or deteriorating symptoms (50%) at the end of follow-up (p > 0.05).

## Discussion

In the current study, we propose that patients with biliary SOD can be managed without SOM. Although it is generally accepted that patients with biliary SOD type I should be treated with biliary sphincterotomy [5,14], there is some controversy as to the management of biliary type II and III patients. There are only two published randomized controlled trials suggesting that biliary sphincterotomy should be performed in patients with biliary SOD type II or III found to have raised basal biliary Sphincter of Oddi pressure upon SOM [15,16]. However, one of the trials only included type II patients [15] whilst the other mainly included type II patients with small numbers of type I and III patients [16] rendering their results hard to generalize to all SOD types. Their findings, moreover, are not in line with uncontrolled studies that failed to show any correlation between SOM results and outcome of sphincterotomy [6,7]. Also, it has been suggested that although abnormally elevated pressure at SOM may predict benefit from biliary sphincterotomy in type II and III patients, the reverse may not hold true [8] and that a single negative SOM study does not rule out sphincter of Oddi dysfunction [17]. Furthermore, Thune et al found that manometric findings compatible with sphincter of Oddi dysfunction, probably due to its episodic nature, are poorly reproducible [18]. A recent study, involving a decision analysis model constructed using a software program, showed that empirical biliary sphincterotomy may be more cost-effective in comparison to a strategy based on the results of manometry for patients with biliary type II pain [19].

In our cohort, a total of 44/59 patients (75%) reported subjective symptomatic benefit after a median follow-up of 15 months. This proportion is similar to that reported in the literature [1-3,16,20-22].

The risk of complications, in particular pancreatitis, is of concern in patients with SOD undergoing ERCP/sphincterotomy [9-11]. The occurrence of post-ERCP complications in our study was 18% in general and 16% for post-ERCP pancreatitis in particular, which is comparable to that in previous reports [1,3,10,22]. Having a post-ERCP complication was not found to have any significant effect on symptomatic relief at follow-up. Placement of temporary prophylactic pancreatic duct stents has been suggested to reduce post-ERCP pancreatitis rates in patients with SOD [23,24]. However, this was not performed in the current study, and thus we cannot exclude the possibility that pancreatic duct stenting might have reduced the incidence of pancreatitis.

There is certain evidence suggesting that the etiology of pain in SOD may be multifactorial. Eighty-five percent of patients continued to have pain at last follow-up with 34% requiring opiate analgesics for pain control (mainly mild opiates). This is in keeping with a previous study in which, despite endoscopic sphincterotomy, 82% of patients with biliary type pain continued to have pain after a mean follow-up of 18 months, indicating that SOD may be a chronic pain disorder with a multifactorial etiology [2]. Duodenal specific visceral hyperalgesia [25] and delayed gastric emptying [3] may be involved in the pathogenesis of pain in these patients. Furthermore, in our cohort 20% of patients experienced at least one recurrence of symptoms after initial response to therapy. Although it has been previously reported that restenosis after endoscopic sphincterotomy for SOD requiring repeated intervention may occur in 13% - 61% during a follow-up of 19 months and 26 months respectively [1,3], ours is the first study to quantify the proportion of patients with biliary type pain who relapse after combined medical and endoscopic management. Further studies, are warranted to fully elucidate the potential mechanisms of abdominal pain in SOD and the role sphincterotomy may play as a component of a comprehensive pain management.

We did not find any differences in response to therapy between the different subclassifications of SOD. This is in keeping with several studies showing that this classification may be inadequate to predict treatment outcome [2,3,20]. It should, however, be noted that a revised version of the initial classification is used nowadays in which noninvasive methods, instead of ERCP, are used to measure CBD diameter and contrast drainage times are no longer utilized [5]. To our knowledge, this revised classification system has not been formally validated.

The presence of an intact gallbladder in our series did not affect symptomatic relief at follow-up. Fifty-six percent of the patients had an intact gallbladder in situ but no gallstones were identified on ultrasound or MRCP/ERCP. Although there are only limited data on SOD and its treatment in patients with gallbladder in situ, SOD is thought to occur in these patients [5]. SOM studies have shown elevated sphincter pressures in a proportion of patients with biliary pain and intact gallbladder [26,27]. Also, endoscopic sphincterotomy has been shown to be effective in patients with biliary-type pain and gallbladder in situ [26,27]. However, radionuclide imaging for gallbladder dyskinesia was not performed in our cohort and thus we cannot exclude that some of the patients with an intact gallbladder might have benefited from cholecystectomy.

Certain methodological limitations should be taken into consideration when interpreting the results of the current study. Although consecutive patients were prospectively identified, part of the data analyzed were retrospectively collected from patient files and no validated questionnaire or structured interview was uniformly used to define treatment outcome. However, all patients were treated in a uniform fashion in a single center under the care of a single consultant gastroenterologist (RWC). Furthermore, discharged patients were not contacted to obtain follow-up data post-discharge. Although we would have expected these patients to have been re-referred to our institution in the event of significant change in symptoms we cannot fully exclude the possibility that some discharged patients who relapsed may have been treated elsewhere. Also, the current study was observational. Large prospective interventional trials enrolling patients with SOD of all types are needed in order to study the most appropriate management approach for these patients. Our data, as well as those published by others [2,3,20], suggest that SOM results may not necessarily define the management course in such future trials.

## Conclusions

In conclusion, patients with biliary sphincter of Oddi disorder may be managed with a combination of endoscopic sphincterotomy (performed in those with dilated common bile duct) and medical therapy without utilization of SOM. The results of this approach with regards to symptomatic relief and ERCP complication rate are comparable to those published in the literature. However, a significant proportion of patients continued to have pain despite subjective symptomatic improvement. Prospective randomized interventional trials enrolling patients with SOD of all types are needed in order to study the most appropriate management approach for these patients.

## Abbreviations

SOD: sphincter of Oddi disorder; CBD: common bile duct; SOML: sphincter of Oddi manometry; ERCP: endoscopic retrograde cholangiopancreatography; MRCP: magnetic resonance cholangiopancreatography; GTN: glyceril trinitrate; IQR: interquartile range

## Competing interests

The authors declare that they have no competing interests.

## Authors' contributions

EK: concept and design, acquisition of data, analysis and interpretation of data, drafting the manuscript, read and approved final manuscript; TA: acquisition of data, critical revision of the manuscript, read and approved final manuscript; JPH: analysis and interpretation of data, critical revision of the manuscript, read and approved final manuscript; JC: analysis and interpretation of data, critical revision of the manuscript, read and approved final manuscript; RC: concept and design, analysis and interpretation of data, critical revision of the manuscript, read and approved final manuscript

## Pre-publication history

The pre-publication history for this paper can be accessed here:

http://www.biomedcentral.com/1471-230X/10/124/prepub
